# The Role of Epigenetics in the Development and Progression of Multiple Myeloma

**DOI:** 10.3390/biomedicines10112767

**Published:** 2022-10-31

**Authors:** Nor Hayati Ismail, Ali Mussa, Nur Atikah Zakaria, Mutaz Jamal Al-Khreisat, Muhamad Aidil Zahidin, Noor Nabila Ramli, Siti Nur Nabeela A’ifah Mohammad, Rosline Hassan, Noor Haslina Mohd Noor, Salfarina Iberahim, Zefarina Zulkafli, Shafini Mohamed Yusoff, Azlan Husin, Muhammad Farid Johan

**Affiliations:** 1 Department of Haematology, School of Medical Sciences, Universiti Sains Malaysia, Kubang Kerian 16150, Kelantan, Malaysia; 2 Department of Biology, Faculty of Education, Omdurman Islamic University, Omdurman P.O. Box 382, Sudan; 3 Department of Internal Medicine, School of Medical Sciences, Universiti Sains Malaysia, Kubang Kerian 16150, Kelantan, Malaysia

**Keywords:** multiple myeloma, DNA methylation, histone modification, ncRNA, epigenetic inhibitors, immuno-oncology

## Abstract

Multiple myeloma (MM) is an exceptionally complicated and heterogeneous disease that is caused by the abnormal proliferation of malignant monoclonal plasma cells initiated in the bone marrow. In disease progression, a multistep process including differentiation, proliferation, and invasion is involved. Despite great improvement in treatment outcomes in recent years due to the substantial discovery of novel therapeutic drugs, MM is still regarded as an incurable disease. Patients with MM are afflicted by confronting remission periods accompanied by relapse or progression outcomes, which inevitably progress to the refractory stage. In this regard, MM may need new medications or modifications in therapeutic strategies to overcome resistance. A variety of genetic abnormalities (e.g., point mutations, translocations, and deletions) and epigenetic changes (e.g., DNA methylation, histone modification, and non-coding RNA) contribute to the pathogenesis and development of MM. Here, we review the significant roles of epigenetic mechanisms in the development and progression of MM. We also highlight epigenetic pathways as potential novel treatment avenues for MM, including their interplay, use of epigenetic inhibitors, and major involvement in immuno-oncology.

## 1. Introduction

Multiple myeloma (MM) is derived from the development of clonal plasma cells (PCs) and the mass synthesis of monoclonal proteins in the bone marrow which eventually leads to end-organ failure [1]. The older population is the most affected by MM, with a median diagnosed age of 69 years [2], which is closely associated with a dismal prognosis and has a 5-year survival rate of 48.5% [3]. It is hypothesised that the MM clone originates from a post-germinal centre as a result of isotope switching and produces a PC that could continue to multiply endlessly [4]. Patients suffering from early-stage MM are diagnosed as having a monoclonal gammopathy of unknown significance (MGUS), a premalignant illness that can proceed to asymptomatic (or smouldering) (SMM) and symptomatic MM [5]. Genetic and epigenetic abnormalities commence at the disease’s outset and continue during the illness, affecting the course of the disease [6].

The microenvironment of bone marrow (BM) is also crucial for MM growth and survival [7]. Interestingly, independent malignant plasma cells develop new abnormalities that allow them to survive outside the BM microenvironment; these cells disperse in the bloodstream or spread to other tissues to develop into more destructive stages, known as plasma cell leukaemia or extramedullary plasmacytomas [1,8]. To date, significant improvement has been achieved in the use of advanced therapeutic agents, including the combination of medications with various modes of action, such as corticosteroids, proteasome inhibitors (PIs), anthracyclines, alkylating agents, monoclonal antibodies (mAbs), immunomodulatory drugs (IMIDs), nuclear export inhibitors, histone deacetylase inhibitors (iHDACs), and heavy doses of chemotherapy drugs followed by a transplant of the patient’s own stem cells (ASCT) [9,10]. However, MM persists as a manageable though critically incurable and lethal disease [11].

## 2. Characterisation of Multiple Myeloma

MM is characterised as an extremely heterogeneous disease due to the intricate genetic alterations that emerge during the progression from the MGUS stage [12] to the SMM stage [3], resulting in various molecularly identified subgroups based on clinical and pathological features [13]. Approximately 1.0% of patients with MGUS acquire active MM each year [14]. As MM progresses, an even more complex evolutionary process and modification of clonal genome architecture is observed in the patients [15]. The overall response and survival rates vary among patients receiving similar treatment because of the large diversity of target candidates for genetic modification and the customised nature of the treatment [16,17,18].

**Figure 1:**
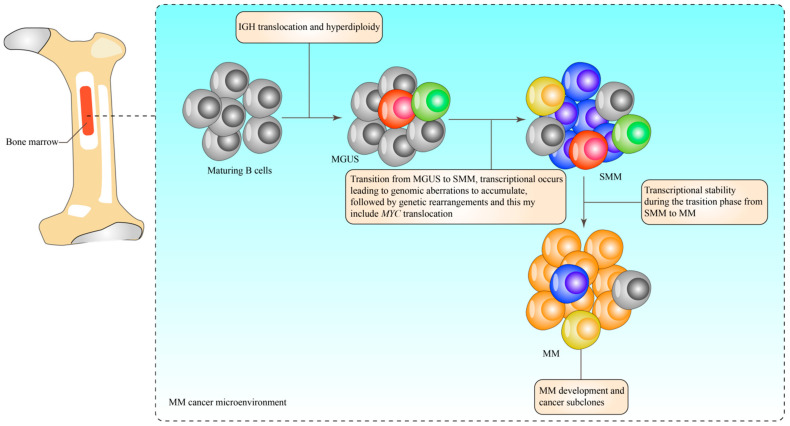
The MM cells’ development in the bone marrow. MM cell development in the bone marrow begins with the maturation of normal B cells, at which point they undergo IGH translocation and produce hyperdiploidy, both of which encourage the progression of the disease to MGUS. In the case of MGUS, cells undergo several transcriptional errors, which results in the accumulation of genomic aberrations. This is then followed by genetic arrangements, such as *MYC* translocation, for the cells to progress to a more cancerous stage, known as SMM. In addition, SMM cell clones establish stability with an incorrect transcriptional rate, which is a prerequisite for the development of cancerous subclones of MM cells. IGH: Immunoglobulin heavy chain; SMM: Smouldering Multiple Myeloma; MGUS: Monoclonal gammopathy of undetermined significance, and MYC: Master Regulator of Cell Cycle Entry and Proliferative Metabolism.

Patients with MM are grouped into two extensive categories, namely, hyperdiploid and non-hyperdiploid, based on genetic and high-risk aberration patterns [13]. A better prognosis is associated with the trisomies of the odd-numbered chromosome of the hyperdiploid group. Vice versa, the established primary translocation events at the immunoglobulin heavy-chain loci (IgH) [19,20] are an indication of poor prognosis [19,21]. Secondary translocations and mutation events are continuously attained throughout the disease progression. A significant variation was observed in the onset to progression to MM due to genetic factors involving del(17p), t(4;14), myelocytomatosis viral oncogene homolog (MYC) translocations [22], gain(1q) [23,24], and gene expression profile (GEP) risk score [25]. Figure 1 illustrates the development of MM cells in the bone marrow.

MM is characterised by epigenetic alterations, which promote clonal heterogeneity and plasticity, thereby contributing to the variety of phenotypes of myeloma-propagating cells and the development of therapy resistance [26]. Recently, extensive research outputs from sequencing and gene expression profiling in MM have revealed multiple epigenetic impairments initiated from DNA hypermethylation and hypomethylation in B cell-specific genes; these phenomena contribute to the tremendous variations in copy numbers that influence aberrations in the expression profiles of several chromatin-modifying enzymes. Emerging research has implicated well-established epigenetic mechanisms (e.g., histone modification patterns and DNA methylation) [27,28] and abnormal microRNA (miRNA) expression [29,30] in MM. Indeed, expanding our knowledge and understanding of this fatal disease will pave the way for effective treatment.

## 3. Epigenetics

Epigenetics is referred to as reversible heritable gene expression modifications without necessitating structural DNA in sequence alterations that might be handed down to an individual’s offspring’s chromosomes [31]. Epigenetic modifications may be the primary starting events in certain cancers [32]. In addition to well-known genetic defects, recent research indicates that aberrant miRNA synthesis, erroneous DNA methylation, abnormal histone modification patterns, and other epigenetic abnormalities may contribute to the pathogenesis of MM. Epigenetic processes play a very crucial role in explaining how and why MM illness has grown with a high degree of clonal heterogeneity and plasticity [28,33].

## 4. DNA Methylation in the Development of Multiple Myeloma

MM presents with global DNA hypomethylation and gene-specific promoter hypermethylation, which are necessary to modulate gene expression during B cell differentiation and maturation [34]. Various cancer-related genes, including p15, p16, p53, p73, E- BNIP3, CAD, CDKN2A, DAPK1, RB1, DIS3, and CDKN2C, are rendered silent as a result of global hypomethylation [35]. The patterns of DNA hypomethylation separate normal PCs from MGUS and MM cells [36,37]. In addition, comprehensive reports of aberrant DNA hypermethylation events in the promoter regions of numerous tumour suppressor genes in MM have been published [38]. Numerous genes that inhibit tumour growth, such as CDKN2B, CDKN2A, CDH1, DAPK1, SOCS1, and SHP1, were rendered inactive as a result of DNA hypermethylation at the CpG islands that are associated with their promoters [39].

Cyclin-dependent kinase (CDKN) with a methylated promoter increases cell proliferation and disease progression by overexpressing CDKs 4 and 6 and has three times the S-phase level observed in 19–53% of patients with MM. Plasma cells in unmethylated patients with CDKN methylation demonstrated a threefold increase in S-phase arrest [40]. In addition, elevated blood levels of beta 2-microglobulin during the progression from MGUS to MM and C-reactive protein tests revealed a poor prognosis for the patient [41]. Moreover, active patients with MM have a higher percentage of E-cadherin (E-CAD) methylation (27–56%) than patients with MGUS, thereby promoting the downregulation of cell adhesion within the tissue and stimulating cellular mobility and metastasis; hence, E-CAD could become an important biomarker for evaluating disease progression [42]. In contrast, the protein kinase (DAPK) gene was shown to be hypermethylated at comparable rates in MM and MGUS, indicating that this event occurred early in the pathogenesis of MM [42,43]. This gene was also associated with a poor response to treatment and a low overall survival (OS) rate [44]. Another study demonstrated that JAK-STAT overactivation via methylation in SH-2-containing phosphatases (SHPs) and suppressors of cytokine signalling (SOCS) genes increased MM survival and proliferative signalling. SHP and SOCS genes had methylation rates of 20–79.4% and 0.0–62.9%, respectively. These methylation events revealed a distinct function, with SHP indicating illness progression and SOCS1 indicating the early onset of MM progression [45].

In myeloma-associated bone diseases, improper epigenetic remodelling of bone marrow mesenchymal stromal cells (MSCs) leads to tumour-promoting behaviour and protracted osteoblast suppression via aberrant transcriptomes (MBD). Extensive degrees of DNA methylation alteration have been reported, specifically in homeobox genes, which culminate in osteogenic differentiation and influence aberrant expression [46]. Demethylation of their promoters alters their expression regulation during osteogenesis [47]. In this perspective, the HOX family has been viewed as the primary driver in osteoblast differentiation [48]. Table 1 describes the gene-targeting DNA methylation events and their effects on MM.

## 5. Histone Modifications in Multiple Myeloma

Post-translational modifications (PTMs) in histone proteins are reversible enzyme-catalysed modifications that mainly consist of methylation, acetylation, phosphorylation, glycosylation, ubiquitylation, SUMOylation, and ADP-ribosylation [66]. The pivotal roles of histone acetylation, methylation, and phosphorylation in controlling gene expression have been addressed throughout the decades as the most described PTMs [67,68].

### 5.1. Histone Methylation

Histones are methylated at all fundamental residues, for instance, at arginine (R), lysine (K), and histidine (H) [69,70]. Arginine occurs in mono (me1), symmetrically dimethylated (me2s), and asymmetrically dimethylated (me2a) forms, whilst lysine exists in mono- (me1), di- (me2), and tri-(me3) methylated forms. Histone H3 lysine 4 (H3K4), H3K9, H3K27, H3K36, H3K79, and H4K20 are the most favourable positions for histone methylation. Conversely, H3R2, H3R8, H3R17, H3R26, and H4R3 have been identified as methylation sites for arginine [69].

### 5.2. Roles of Histone Methylation Modifiers in Multiple Myeloma

Histone methyltransferases (HMTs) are a class of enzymes that catalyse the methylation of lysine and arginine residues. Histone methylation at arginine residues occurs at their guanidinium side chains and is catalysed by protein arginine methyltransferases (PRMTs). PRMT4 and PRMT5 have been identified as targets in MM due to their prognostic importance. Coactivator-associated arginine methyltransferase 1 (CARM1) serves as a transcriptional activator that facilitates the methylation of non-histone proteins and H3R2me2a, H3R17me2a, and H3R26me2a [66]. The overexpression of CARM1 was reported to have a role in MM; a potent CARM1 inhibitor, namely, EZM2302, significantly inhibited the growth of MM tumour [67]. The methyltransferase activity of PRMT4 was strongly suppressed by TP-064, another well-known CARM1 inhibitor that has higher selectivity than other PRMTs [68]. EZM2302 and TP-064 are strong drug candidates for the application of the CARM1 inhibitor in clinical settings to prevent MM progression.

PRMT5 was overexpressed in CD138+ immunopurified cells derived from patients with MM, and the upregulation of PRMT5 was closely related to the decreased OS and progression-free survival rates [71]. A PRMT5 inhibitor that is orally accessible, namely, EPZ015666, was examined and found to dramatically reduce the growth of both cell lines and patient MM cells. Thus, EPZ015666 could be a novel therapy for MM [71]. In addition to the upregulation of PRMT5 expression in MM, PRMT5 was also recently discovered to regulate cell pyroptosis by negative correlation to CASP1 (a gene that encodes caspase 1) in MM, by which, PRMT5 activity inhibition enhanced the CASP1 expression [72]. This new finding helps to understand the underlying mechanisms in MM development and has also been linked to CASP1-mediated cell pyroptosis [73].

The methylation of histone H3K36me3 is catalysed by the nuclear receptor binding SET domain (NSD) family, which is composed of enzymes NSD1, NSD2/multiple myeloma SET domain (MMSET)/Wolf–Hirschhorn syndrome candidate 1 (WHSC1) and NSD3/WHSC1L1 [74]. MMSET/NSD2 is an oncoprotein that is aberrantly expressed in MM that leads to abnormally high levels of H3K36 dimethylation and affects the zeste homolog 2 (EZH2) enhancers; this protein significantly decreases H3K27me3 methylation and promotes malignant cell growth, adhesion, and chromatin accessibility [75,76]. EZH2 expression is considerably increased as MM develops from MGUS and SMM, peaking at the plasma cell leukaemia (PCL) stage [77]. Alternative splicing and transcriptional elongation are two processes that have been linked to the HMT called SETD2, which has also been shown to tri-methylate H3K36. In MM cell lines that carry the t(4;14) translocation, inhibiting SETD2 reduced the global tri-methylation of H3K36, which in turn led to a decrease in the lines’ capacity for proliferation [75].

Histone lysine demethylases control the demethylation of the lysine residues (KDMs). KDM1A and KDM1B perform functions as co-factors in demethylation reactions that include H3K4me1/2 or H3K9me1/2 residues [78]. The remaining KDM members each have a domain known as Jumonji C (JmjC) and are co-factored by oxygenases that are dependent on either iron (II) or 2-oxoglutarate (2-OG) (2OGXs). KDM3A upregulation by removing the H3K9 methyl marks directly increases the expression of Kruppel-like factor 2 (KLF2) and interferon regulatory factor 4 (IRF4), indicating that the KDM3A-KLF2-IRF4 axis plays a biological role for KDM3A in the pathogenesis of MM cancer. This finding was discovered in a study on MM cancer, where KDM3A knockdown was found to be toxic to MM cells [79]. The research established a strong oncogenic significance for the HIF-1-KDM3A-MALAT1 axis positive feedback loop in MM. HIF-1, a transcription factor that reacts to an environment that supports cancer and is hypoxic, was necessary for KDM3A to function. Through KDM3A-mediated positive feedback, MALAT1 (long noncoding RNA) expression is elevated and subsequently promotes glycolytic gene expression, leading to MM cell apoptosis [80].

KDM6A mutations or deletions are frequent characteristics of MM cell lines and speed up the course of the disease by enhancing MM cell proliferation, clonogenicity, adhesion, and tumorigenicity [81]. A KDM6A deficit is significantly positively correlated with the expression of transcriptional regulators of the Major Histocompatibility Complex I and II (MHCII and MCHII), as well as NLRC5 (NOD-like receptor family CARD domain containing 5) and CIITA (Class II transactivator). KDM6A deficiency may help MM cells to escape immune recognition and therefore promotes the systematic spread of the tumour, explaining the MM progression in patients [81]. Furthermore, KDM6B was extensively expressed in MM to the point where its absence results in the death of MM cells. KDM6B recruits to the loci of genes encoding components of the MAPK signalling pathway and upregulates the expression of these genes without affecting H3K27 methylation levels, regulating the mitogen-activated protein kinase (MAPK) pathway molecules in MM cells in a demethylase-independent manner. This discovery was made possible by the fact that KDM6B can upregulate the expression [82].

### 5.3. Histone Acetylation

Histone acetylation is generated by enzymes known as histone acetyl transferases (HATs) and acetylation activity is opposed by histone deacetylase (HDACs) [83]. Hyperacetylated chromatin leads to an open, transcriptionally active state due to the repulsive force between neighbouring histones and the DNA, thus exposing the specific gene to the transcriptional machinery. Removal of acetyl groups by HDACs leads to the closed state of the chromatin, suppressing the transcriptional activity [84].

### 5.4. Roles of Histone Acetylation Modifiers in Multiple Myeloma

The proliferation and survival of MM are dependent on the activity of CBP/p300, where CBP/p300 inhibition could lead to the direct suppression of IRF4 expression and concomitantly, IRF4 suppress the oncogenic transcription factor c-MYC, thus providing anti-myeloma effects [85]. These data suggest that targeting the oncogenic transcription networks could be a promising therapeutic strategy for MM cancer.

Clarification of the carcinogenic significance of these three proteins was achieved in MM cells by inhibiting their expression of HDAC1, HDAC2, and HDAC3, respectively. It was found that among these three isoforms, HDAC3 plays the most important role in MM cell proliferation and survival, whereas HDAC1 and HDAC2 knockdown have minimal to no inhibitory effects in MM cells [86]. Not limited to the direct effect of HDAC3 knockdown on MM cells, HDAC3 knockdown in the bone marrow microenvironment also has indirect effects on MM cells by limiting MM cell growth and survival [87]. HDAC4 expression is also upregulated in MM significantly, to the extent that its knockdown suppresses the MM cells’ growth and triggered apoptosis and autophagy. Interestingly, under endoplasmic reticulum stress conditions due to the accumulation of unfolded protein in MM, HDAC4 inhibition increased activating transcription factor 4 (ATF4) expression and was associated with MM cells’ cytotoxicity and apoptosis [88]. Epi-miRNA, miR-29b, was demonstrated to have antagonistic effects on HDAC4 overexpression in MM previously, suggesting its potential to eradicate MM cells [89].

HDAC6 is involved in the aggregation of misfolded proteins in the cell to form aggresomes [90]. HDAC6 has a mutually reinforcing relationship with c-Myc in which the knockdown of c-Myc reduces HDAC6 expression [91]. A member of class III HDACs, sirtuin 6 (SIRT6), is significantly expressed in MM cells and leads to the acceleration of disease progression. SIRT6 knockout MM cell lines downregulate the transcription of DNA-damaged genes, thus enhancing anti-MM activities and proving that SIRT6 is a good candidate for MM therapeutic target [92]. Class IV HDAC, HDAC11, is responsible for B cells’ maturation into plasma cells through crosstalk with IRF4. Inhibition of HDAC11 activity results in the hyperacetylation of IRF4 and subsequently induces cytotoxicity in MM cells as well as impairment of plasma cell development [93]. Taken together, the inhibition of HDACs activities by various HDACs inhibitors serves as a great strategy to tackle the MM cells’ aggressive growth and induces apoptotic activity on MM cells, thus minimising the MM disease progression in patients.

**Figure 2:**
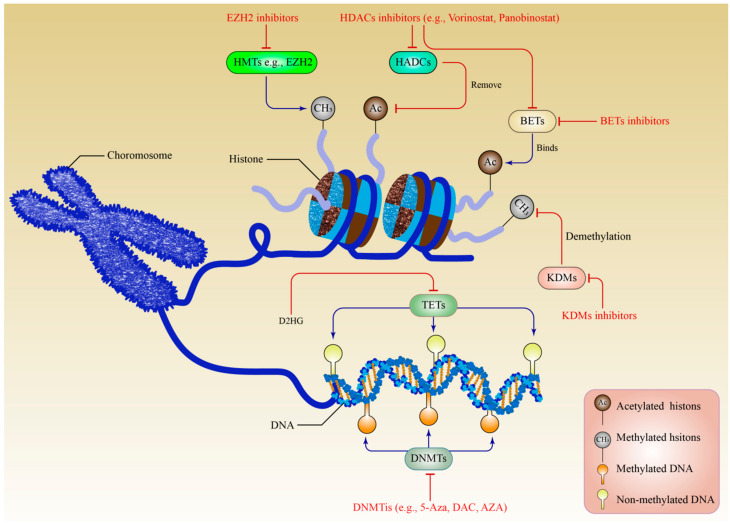
The methylation pattern in MM development. Both EZH2 inhibitors and HDACi (vorinostat and panobinostat) remove the Ac group which is attached to the transcriptional tail that is exposed to the histone body to make it possible for transcription to occur. EZH2 inhibitors inhibit the activity of HMTs by removing the CH3 group. Additionally, HDACi focuses on the BET domain since BET proteins physically connect to the enhancer and promoter regions to increase the beginning and continuation of gene transcription. Additionally, BET inhibitors have been shown to have an anti-MM effect in vitro and/or in vivo by blocking the gene expression network of the MM. The demethylation process was mediated by two groups of KDMs, and the presence of KDMi resulted in a decreased expression of the mechanism. The epigenetic alteration that results from increased levels of D2HG relates to changes in the pattern of gene expression. This suppression of the TETs family of DNA demethylases is caused by elevated levels of D2HG. Therefore, DNMT is damaged and passive DNA demethylation occurs when DNMT inhibitors such as 5-Aza, DAC, and AZA integrate into DNA. This process blocks the covalently bound DNMT enzymes and results in DNMT damage. EZH2: Enhancer of zeste homolog 2; HDAC: Histone deacetylases; HDACi: Histone deacetylases inhibitor; Ac: Acetyl; CH3: Methyl; HMT: Histone methyltransferases; BET: bromodomain and extra-terminal; KDMs: Histone lysine demethylases; KDMi: Histone lysine demethylases inhibitor; D2HG: D-2-hydroxyglutarate; DNMT: DNA methyltransferases; 5-Aza: 5-azacytidine; DAC: Decitabine; and AZA: Azacytidine.

DNA methylation and histone PTMs, which are the two primary epigenetic processes in MM, were seen to interact to a significant degree. DNA methylation enzymes are responsible for determining how the histone modification process plays out and the effects it produces. As a result, double epigenetic modulation targeting both DNMTs’ epigenetic processes and HDAC inhibitors was used in refractory and poor-risk relapsed lymphoma patients to improve the efficacy of high-dose chemotherapy in these patients [94]. Figure 2 describes the epigenetic process involving both DNA methylation and histone modification mechanisms in MM.

## 6. Importance of Non-Coding RNA in MM

miRNAs exhibit a crucial role in controlling the pathogenic elements such immunomodulation, the tumour microenvironment, DNA methylation, genomic instability, and treatment resistance that contribute to the development of MM. Additionally, they might be used as possible therapeutic targets and prognostic biomarkers to play a critical role in the management of treatment resistance by targeting oncogenes and tumour suppressor genes in neoplastic disorders [95]. The expression levels of certain microRNAs, such as miR-125b, miR-133a, miR-1, miR-124a, miR-15, and miR-16, are lower in the cell samples of patients with MM [96,97]. In addition, MM plasma cells had higher levels of miR-21 expression than plasma cells from patients without MM [98]. Some studies showed that miR-203 has an 83% greater specificity and sensitivity in the diagnosis of MM [99]. The levels of the microRNAs miR-21, miR-221/222, miR-125a and miR-125b, and miR-451 are all elevated in MM cells that are resistant to anti-myeloma treatments [100,101,102]. miR-1246 expression was significantly higher in patients with MM, regardless of age, gender, stage, blood levels of β2-microglobulin, albumin, calcium, creatinine, myeloma protein, and haemoglobin, as well as the population of bone marrow plasma cells and chromosome 13 deletions. Based on this information, miR-1246 has the potential to be an essential biomarker for the diagnosis of MM [103]. In addition, Bong et al. [104] found that the microRNAs miR-150 and miR-125b are uniquely connected to the development of B cells. While miR-125b may target the RAS and CysLT signalling proteins RASGRF2 and CYSLTR2, miR-150 may function as a negative regulator for the genes RAD54L and CCNA2, which are essential for cell cycle regulation [104].

It is conceivable that an abnormality in miR-21 has a role in the preliminary phase of piRNAs and makes up 20 to 30% of the total RNA in exosomes. Maintaining the functions of germ and stem cells requires piRNAs, which range in length from 24 to 32 nucleotides. They accomplish this by controlling epigenetics and preserving genetic stability in germlines [105,106]. PiRNA-823 directly recruited DNMT3a and DNMT3b into primary CD138+ MM cells, therefore increasing overall DNA methylation and inhibiting the expression of the tumour suppressor p16INK4A [107].

In addition, lncRNAs have been found to recruit chromatin-modifying proteins, thus managing the interactions between distal regulatory elements or creating long-range chromosomal regulatory domains and nuclear bodies [108], therefore influencing gene expression in the post-transcriptional phase [109,110]. Four lncRNAs, including RP4-803 J11.2, RP1-43E13.4, RP11-553 L6.5, and ZFY-AS1, showed prognosis value and a high correlation with OS in patients with MM. The fact that lncRNA is involved in chromatin modification, DNA replication, DNA repair, and RNA processing lends credence to this idea [111], which in turn supports the crucial role that lncRNA plays in genomic and epigenetic events throughout the development of MM. Even though several studies have established the role of lncRNA in cellular homeostasis, many of its fractions remain unexplored, necessitating more research.

## 7. Targeting Epigenetic Mechanisms as Novel Treatment Modalities in MM

### 7.1. Targeting DNA Methylation

Between the years 2004 and 2015, the United States Food and Drug Administration (FDA) granted authorisation for the use of six different epigenetic agents in clinical trials. These epigenetic agents include azacytidine, 5-aza-2′-deoxycytidine, suberoylanilide hydroxamic acid (SAHA), romidepsin, belinostat, panobinostat and chidamide [112]. In addition, the FDA has granted approval for the therapeutic use of 5-aza-2′deoxycytidine (5-aza-AZA; decitabine) and 5-aza-acytidine (5-aza-AZA) for the treatment of patients who suffer from MDS and chronic myelomonocytic leukaemia. These two epigenetic drugs did not have a clinical license to treat myelodysplastic syndromes [113], but they did display anti-myeloma efficacy in vitro and in vivo [114,115]. AZA [116] and DAC [117] accelerate clonal cell cycle arrest by boosting the activation of negative cell cycle regulators, which ultimately leads to apoptosis and senescence pathways (p16 and p15). G0/G1 and G2/M cell cycle arrests involving p21 and p38 were detected following DAC therapy [117]. Curiously, AZA in combination with doxorubicin and bortezomib had synergistic anti-MM efficacy [118] and restored sensitivity to dexamethasone [119]. Both AZA and 5-aza-2′-deoxycytidine (DAC) are capable of exerting detrimental effects by integrating into DNA and blocking covalently bound DNMT enzymes, which in turn causes DNMT damage and passive DNA demethylation [120]. In a model of murine myeloma, treatment with CM-272, an inhibitor combination that blocks both DNMTs and the histone methyltransferase G9a, decreases bone loss associated with the tumour and reduces the overall volume of the tumour. Utilising inhibitors allows for the induction of osteoblast formation in myeloma MSCs and the restoration of the expression of hypermethylated osteogenic regulators [46].

### 7.2. Targeting Histone Acetylation

HDACi displays anti-MM activity in cells by activating the apoptotic pathway, inhibiting the proteasome, and decreasing tumorigenesis and treatment resistance [121,122]. WT161, an HDAC6 inhibitor, induces cell death by boosting tubulin acetylation and inhibiting aggresome-dependent protein degradation in MM cells both in vitro and in vivo [123]. Panobinostat (LBH589) is a pan-HDACi that interacts with bortezomib and has been licensed for patients with relapsed or refractory MM [124] due to its ability to inhibit class I, II, and IV HDACs at a low nanomolar concentration [125,126]. This agent inhibits aggresome and proteasome networks and enhances the acetylation of proteins implicated in many carcinogenic pathways in MM cells [127]. Thus, progression-free survival (PFS) and complete and near-complete responses were significantly enhanced. Nevertheless, some individuals had adverse symptoms, including thrombocytopenia, diarrhoea, asthenia, and weariness. The combined treatment of panobinostat, bortezomib, and dexamethasone is predicted to benefit patients with MM cancer who developed bortezomib resistance [128]. Additionally, the first oral selective HDAC6 inhibitor, Ricolinostat (ACY-1215), showed reduced class I HDAC activity when coupled with carfilzomib [129], lenalidomide, and dexamethasone [130], showing anti-MM effects following therapy. HDACi concentrates on bromodomain and the extra-terminal domain (BET) because the BET proteins physically link the enhancer and promoter regions to stimulate the initiation and extension of gene transcription. By blocking the MYC oncogene and its gene expression network, BET inhibitors have shown an anti-MM effect in vitro and/or in vivo, either alone or in combination, highlighting that BET inhibitors might be considered a feasible therapeutic intervention in MM [131].

### 7.3. Targeting Histone Methylation

Inhibitors of histone methyltransferase for enhancer of zeste homolog 2 (EZH2) are emerging as an epigenetic therapy strategy for MM disease whether used alone or in combination with other targeted drugs. EZH2, which contains the enzyme component of the polycomb repressive complex 2 (PRC2), is essential for both normal cell development and the progression of disease (PRC2). PRC2 in EZH2 catalyses the methylation of histone H3 lysine tail residue 27 (H3K27me3), which induces the reprogramming of cells associated with stem cell self-renewal, cell cycle, cell differentiation, and cellular transformation. Thus, the discovery of highly selective inhibitors of EZH2’s histone methyltransferase activity has shed light on the function of EZH2 and PRC2 in carcinogenesis and their potential as cancer therapy targets [77].

Since both target combinations allow for the control of gene expression, histone H3 lysine 27 (H3K27) methyltransferase and G9, an H3K9 methyltransferase, have been identified as another promising therapeutic target in MM. To be more specific, the combination of these two inhibitors induces cell cycle arrest and triggers the pathway that leads to apoptosis, which in turn lowers the rate of MM cell growth. In addition, an examination in animals demonstrated an anticancer effect, as shown by a decrease in the formation of MM cell xenografts. There is also a correlation between greater levels of EZH2 and EHMT2 expression (both of which encode G9a) and worse outcomes for patients with MM. In contrast, the inhibition of EZH2/G9a resulted in an increase in the expression of genes that are activated by IFN and a reduction in the expression of genes that are involved in the IRF4-MYC axis in MM cells. This is supported by the observation that the degree of ERV gene expression in MM cells has dramatically risen and that the H3K27/H3K9 methylation levels have decreased, both of which are indicators of an IFN response [132].

The methylation process in histone H3 lysine-4 (H3K4), -36 (H3K36), and -79 (H3K79) caused transcriptional pathway upregulation in MM cells. Gene silencing events, on the other hand, were shown by methylation involving histone H4 lysine 20 (H4K20) [133]. For instance, GSK126, the EZH2 inhibitor, has been administrated to patients with MM with relapsed or refractory phases in phase I clinical trials [134]. Furthermore, MMSET histone methyltransferase was discovered as a promising target for epigenetic treatment in MM due to the anti-tumour activity shown following the shRNA-mediated suppression of MM cells in vitro and in vivo [135]. Consequently, LEM-06 has been introduced as an MMSET inhibitor to serve as an alternative model for assessing the therapeutic potential of MMSET in MM [136].

Table 2 outlines the types of epigenetic inhibitors administered to patients with MM, along with their mode of action.

### 7.4. Targeting MicroRNAs in MM

miRNAs have been suggested as a new class of agents for MM therapeutic intervention since extensive research indicated their deregulation effect on MM cells and the possible targeting of several oncogenes or tumour suppressor genes, hence modifying MM development in vitro and in vivo [157]. Dysregulation of the tumour suppressor miRNA, miR-155, leads to the suppression of MM cell proliferation and treatment resistance. miR-155 overexpression increased drug-resistant MM cells’ sensitivity to bortezomib in a dosage and time-dependent manner. miRNA-155, on the other hand, targets TNF-mediated apoptosis by decreasing caspase-8 activity, inhibiting BID cleavage and caspase-3 activation [158,159]. Furthermore, the potentiality of this inhibitor to reduce CD47 on the cell surface activates the phagocytosis process through macrophage activity, resulting in tumour regression and enhanced bone resorption in animal models [160].

The hsa_circRNA_101237 has been investigated as a potential candidate for a circular RNA (circRNA) diagnostic biomarker for multiple myeloma (MM) malignancy. When hsa_circRNA_101237 was upregulated, it was discovered that a few signalling pathways such as PI3K-Akt signalling and chemokine signalling, that are cell cycle-related, as well as the signalling pathway of cytokines and their receptors were affected. These complex interactions are associated with significantly reduced OS and PFS rates [161]. Patients with MM who received four cycles of bortezomib-containing therapy had a differential response, with a higher M protein decrease correlating with a declined expression of hsa_circRNA_101237 [161]. This is observed in contrast to patients with MM who received treatment that did not contain bortezomib. Patients who received treatment that did not include bortezomib did not have a differential response. In addition, individuals who had a deletion of 13q14, an amplification of 1q21, a deletion of the P53 gene, and mutations in the t(4,14), t(14,16), and t(11,14) genes were observed to have overexpression, but mutations in the t(11,14) gene had the opposite effect. Overexpression has a substantial effect on the prognosis of patients with MM. In addition to this, patients who had multiple myeloma that relapsed or was resistant to treatment exhibited higher expression of the hsa_circRNA_101237 [161].

**Figure 3:**
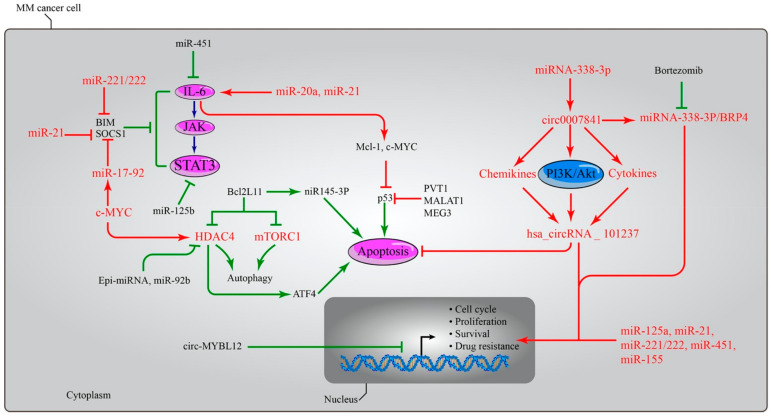
The dual roles of microRNAs in MM. microRNAs have the capacity to influence both the expressions of oncogenes and tumour suppressor genes. The upregulation of miR-21, miR-221/222, and miR-17-92 causes the deactivation of the BIM, SOCS1, and IL6-JAK-STAT3 pathways, which results in the activation of the Mcl-1, Bcl-XL, and c-Myc oncogenes and the suppression of the activity of the p53 gene through the LncRNAs PTV1, MALAT1, and MEG. In other pathways, the activation of miIR145-3p led to increased rates of the pro-apoptotic protein BCL2L11, and the inactivation of mTORC1 led to activation of the autophagic flux, which in turn led to increased autophagy and cell death. Both effects were caused by an increase in the rate of autophagy. miRNA-338-3p served as a substrate for circ_000784, which in turn activated the PI3K/AKT signalling pathway, as well as chemokines and cytokines, ultimately leading to the activation of hsa_circRNA_101237. Circ_0007841 revealed its oncogene activity via the miRNA-338-3p/BRD4 complex to boost cell proliferation and the processes of the cell cycle, hence bypassing the cell apoptosis and senescence pathway. Patients with multiple myeloma had significantly decreased levels of circ-MYBL2 expression in both bone marrow and serum, which is indicative of a poor prognosis owing to the advanced clinical stage of their disease and other variables. However, treatment with exogenous circ-MYBL2 resulted in a significant increase in the rate at which MM cells succumbed, as well as improved DNA synthesis and machinery for proliferation. Overexpression of miR-155 led to a dose- and time-dependent increase in drug-resistant multiple myeloma cells’ sensitivity to the anticancer drug bortezomib. In addition, the levels of miR-125a, miR-21, miR-221/222, miR-451, and miR-155 are increased in MM cells, which boosts the cell cycle, cell proliferation, and survival rate, as well as the likelihood of developing resistance to anti-myeloma therapy. miR: microRNA, BIM: Bcl-2-like protein 11, SOCS: Suppressors of cytokine signalling, IL6: Interleukin-6, JAK: Janus kinase, STAT: Signal transducer and activator of transcription, p53: tumour protein p53, Mcl-1: Myeloid cell leukemia-1, Bcl-XL: B-cell lymphoma-extra-large, c-Myc: c-myelocytomatosis oncogene product, mTOR: mammalian target of rapamycin complex 1, LncRNAs: Long Non-coding RNA, PTV1: plasmacytoma variant translocation 1, MALAT1: metastasis-associated lung adenocarcinoma transcript 1, MEG: maternally expressed gene, BCL2L11: Bcl-2-like protein 11, PI3K/AKT: Phosphatidylinositol-3-Kinase and Protein Kinase B, and BRD4: Bromodomain Containing 4.

Additionally, miR145-3p inhibits the growth of MM cells by initiating the apoptotic pathway in vitro and in vivo experiments. Increased rates of the pro-apoptotic protein BCL2L11 and the inactivation of mTORC1 result in the activation of the autophagic flux, which in turn causes increased autophagy and cell death [162]. Furthermore, a significantly lower expression of circ-MYBL2 was indicated in MM bone marrow and serum [163], indicating a poor prognosis due to their advanced clinical stage and other factors. Treatment with exogenous circ-MYBL2 resulted in a robust MM cell death rate and enhanced DNA production and proliferation machinery. In a manner analogous to this, the treatment of circ-MYBL2 has been shown to inhibit the growth of subcutaneous xenograft tumours in experimental animal models [164]. Additionally, miRNA-338-3p was a substrate to circ_000784, and both miRNA-338-3p and circ_0007841 showed anti-MM effects on the development and progression of multiple myeloma. Because of the efforts made by miRNA-338-3p, the PI3K/AKT signalling pathway was made more effective by circular 0007841 [162,165]. Circ_0007841 displayed its oncogene activity through the miRNA-338-3p/BRD4 complex to increase cell proliferation and cell cycle mechanisms, eventually evading the cell apoptosis and senescence route [165,166]. The effects of ncRNAs on the expression of oncogenes and tumour suppressor genes in MM are illustrated in Figure 3.

## 8. Epigenetic Effects on Immunomodulation in MM

Epigenetic modulators have triggered the reactivation of anti-tumour responses by increasing immune recognition and immunogenicity, and restoring the cell’s immunological tolerant state [167]. After being activated, cyclin-dependent kinases 4 and 6 (CDK4 and CDK6) transform into complexes of cyclin D molecules (D1, D2, and D3), which then induce phosphorylation and cause the Rb (retinoblastoma) protein to become inactive. Therefore, the cell cycle phase transitions to the S1 phase as a result of a prior event that triggered the activation of the CDK2/Cyclin A/E complex associated with the E2F family members. This occurs because the cell cycle phase had previously been in the G1 phase.

In conjunction with other members of the INK4 family, cyclin-dependent kinase inhibitors, often known as CKIs, could block CDK4/6 activity (p16INK4a encoded by CDKN2A, p19INK4d encoded by CDKN2D, p18INK4c encoded by CDKN2C, and p15INK4b encoded by CDKN2B). During this period, it was determined that CDK2 had been inactive because of an inhibitory mechanism involving members of the Cip/Kip family. This mechanism was responsible for CDK2’s inactivity (p57Kip2 encoded by CDKN1C, p21Cip1 encoded by CDKN1A, and p27Kip1 encoded by CDKN1B). Both CDKN2A gene’s coding frames, INK4A and ARF (alternative reading frame), encode for the proteins p16INK4a and p14ARF, respectively. These proteins coordinate with MDM2 to restore the equilibrium of p53 and arrest the cell cycle by inhibiting p21/CDK2/cyclin E [168]. p16INK4a is the most extensively studied cancer gene and has been discovered to be heterogeneous in a multitude of human cancer cell lines as well as primary tumours [169]. This heterogeneity is noticeable in regard to point mutations, and it extends to deletion profiles and epigenetic silencing effects as well. On the other hand, an abnormal alteration in this regulatory system causes a diffused overexpression in cyclin D members and CDK4, as well as a loss of function in Rb, which ultimately leads to the advancement of tumours [170].

Overexpression of cyclin D members cyclin D1 and cyclin D3 in 11q13 and 6p21, respectively, in patients with MM, in conjunction with translocations of the IGH locus on 14q32, suggested that MM pathogenesis had commenced [171]. The miR-29b and miR-34 families have been shown to be responsible for the rise of the cyclin D/CDK4/6 complex’s activity. However, the hypermethylation that supposedly occurred in the mIR34B/C promoter caused a decline in the expression of miR-34b/c, something which is often seen in the evolution of the MM disease at its final stage [172]. In addition, the overexpression of the miR-17–92 cluster, which targeted E2F1, was shown to drive the progression of multiple myeloma [173]. Around 40% of newly diagnosed patients with MM displayed hypermethylation at the INK4A promoter, 10–80% at the CDKN2B promoter, and rare incidences of promoter methylation occurred at CDKN2C or members of the Cip/Kip family [174].

Wnt pathways have been discovered to upregulate the transcription process of several genes in MM. These genes include CCDN1 (encoding cyclin D1), STAT3, MYC, and catenin/transforming growth factor (TGF)/LEF1. After the activation of this route, β-catenin is deposited in the intranuclear domain of the cells, where it will then bind to T-cell factor/lymphoid enhancer factor 1 (LEF1) to create a transcription factor complex. This complex will then activate the gene transcription pathways. In MM, the β-catenin/TGF/LEF complex works to decrease the actions of p16INK4a and miR-15a/16. This phenomenon, in turn, aids in the regulation of cyclin D1 and promotes angiogenesis in multiple myeloma [96,175,176]. The secreted Frizzled-related protein (sFRP) and the Dickkopf (DKK) class have both been discovered as protein subclasses that suppress the Wnt pathway, where LRP5/6 is responsible for the identification of these two protein subclasses [164]. It is interesting to note that DKK1 has become an interest in the condition known as myeloma bone disease [177]. Wnt pathway inhibitors include miR-34a [178], miR-203 [179], miR-21, and miR-200a [180], HDAC1, HDAC2, and HDAC, whereas HDAC3 and HDAC6 upregulate Wnt activity [181]. As a consequence, a high burden of tumour invasion and metastasis is related to a deletion in the CDH1 gene, which codes for ECAD [182]. It is hypothesised that the methylation of CDH1 accounts for the progression of MGUS to MM, which is shown by 27–92% of patients with MM. On the other hand, MM and plasma cell leukaemia were shown to have a higher proportion of CDH1 that was hypermethylated [182].

Crosstalk has been reported between the IL-6/JAK/STAT signalling pathway in MM by demonstrating an increased production of IL-6, which was driven by the activation of pathways including the IL-6 receptor and the Janus kinase/signal transducer and activator of the transcription (JAK/STAT3) pathway. Consequently, protein tyrosine phosphatases, the SOCS protein family, and the PIAS (protein inhibitor of activated STAT) protein family have all been demonstrated to have the potential to block the JAK/STAT3 pathway. There was a difference in the rate of hypermethylation that was identified in the promoters SHP1, SOCS1, and SOCS3 [183]; the increased expression of JAK/STAT signalling in MM may be attributed to the fact that SOCS1 and SOCS3 are targets for the miR-17–92 cluster. In addition, PIAS3 acts as a target for miR-21, which in turn encourages JAK/STAT3 activation, which ultimately causes MM cells to circumvent the apoptotic pathway [98,184,185].

Knockdown of death-associated protein kinase (DAPk) is a regular phenomenon seen in tumour cells. This phenomenon suggests that tumour cells have found a means to evade the apoptotic pathway. In a normal cell, DAPk works in conjunction with the p53 gene to induce the overexpression of p14ARF, which ultimately results in an arrest of the cell cycle and apoptosis [186]. Conversely, in MM, it is a typical feature to see hypermethylation in the DAPk promoter and vice versa with the p53 gene. In addition to miR-192, miR-194, and miR-215, the miR-106b-25 cluster was also able to have some influence on the regulation of MM p53 [173,187].

The use of epigenetic inhibitors in combination with immunotherapy has been the subject of extensive research and has been shown to be very effective. DNMT interferes with the DNA methylation process, which in turn restores the function of dormant suppressor genes including p15, P16, MLH1, and RB52 that had been inactive by suppressing the hypermethylation event that occurs in the promoter region. DNMT inhibitors reduce the anti-MM effect by enhancing the immunomodulatory action via a variety of complex pathways. Cancer cells were shown to have an upregulated level of major histocompatibility complex (MHC) I and MHCII. MHC I and MHCII complexes function as a substrate for peptides, which then form complexes with T-cells to boost the immune system. MHCI expression was shown to be elevated in tumour sections taken from mice treated with azacytidine [188]. Furthermore, enhanced immunogenicity and the synthesis of essential immunostimulatory cytokines restored the activity, endurance, and growth of natural killer (NK) cells when combined with T-cells to combat cancer. The stimulation of NK and T-cells results in IFN synthesis and reactivates the CD4 T-helper cell activity. The high methylation rate of IFN-observed in CD8+ T-cells has suggested Demi as a promising insight in treating MM [188]. Decitabine therapy has been shown to be a significant use of DNMT inhibitors since it inhibits the rate of DNA hypermethylation in human leukocyte class I antigens, such as tapasin, TAP1, and TAP2 to reinstate the upregulation of these molecules [189]. In other research, azacytidine was shown to be linked with greater expression levels of immune-related genes in a variety of solid tumours. This suggests that an improved understanding of the role of epigenetics in the treatment of immune disorders will result from epigenetic translation [190]. The restoration of several antigens that are involved in the process of tumorigenesis, such as MAGE, SSX, SPANX, and PAGE, results in an improvement in the immunogenicity of patients [191].

DNMT inhibitors have been linked to decreased immunosuppression in MDS patients by decreasing Treg activity [192]. Decitabine, on the other hand, downregulates MSDCs in a murine ovarian model [193]. In addition, when coupled with anti-CTLA4, it leads to an increase in cytokine production. Consequently, the generation of NK and CD8 cytotoxic T-cells increases, stimulating IFN- and TNF production [190]. Decitabine stimulates PD-1, PD-L1, PD-L2, and CTLA-4 activity, as in leukaemia. In contrast, there is a higher possibility that the overexpression of PD-1 is also associated with resistance to hypomethylating drugs in the group that is resistant to DNMT inhibitors [194]. In addition, azacytidine promotes the demethylation of the PD-1 promoter with research finding higher PD-1 mRNA and a poorer prognosis in MDS patients [195]. Additionally, azacytidine is responsible for the induction of the expression of PD-L1, the higher level of which was seen in NSCLC cell lines during both the transcription phase and the cell’s surface. The most significant benefit of azacytidine, an epigenetic inhibitor, is the restoration of a signalling pathway related to boosting the immune system to raise the number of T-cells, which subsequently destroys tumour cells through a cytotoxic process [196].

Common manifestations of action underlie both the viral protection route and DNMT inhibitor enhancement of the immunological signalling pathway. By misleading cancer cells into pretending they are infected with a virus, the DNMT inhibitor triggers an interferon response in ovarian cancer [197,198]. Lymphocytes were activated in the tumour microenvironment and were able to kill off the malignant cells [199]. In contrast, patients with melanoma who received anti-CTLA-4 immune checkpoint medication had a much better response and longer survival time [198]. Other research on colon cancer found that EZH2 and PRC2 mediated the suppression of Th1-type chemokines CXCL9 and CXCL10 expression and production. The PRC2 apparatus was anticipated to have a negative correlation with CD4^+^, CD8^+^, and Th1-type chemokines, and this connection was thought to be substantially related to the prognosis of patients [200].

**Figure 4:**
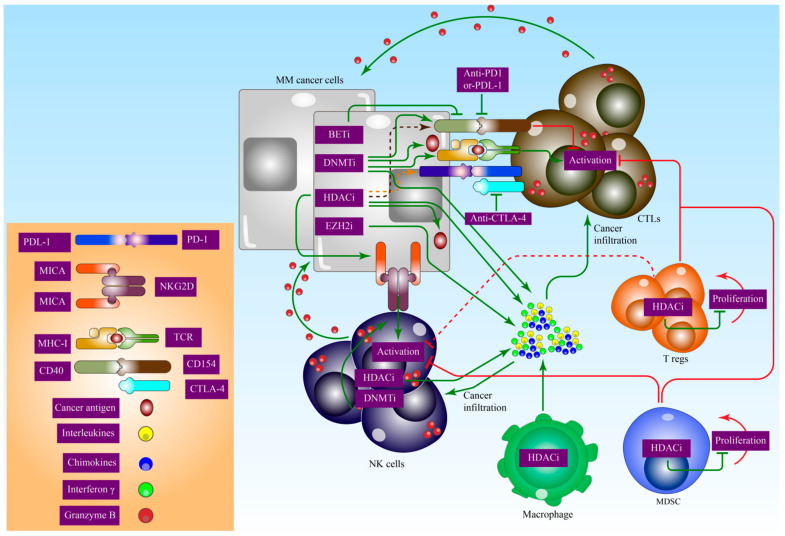
Epigenetic inhibitors also have an important role in MM immuno-oncology. Three classes of epigenetic inhibitors are currently in clinical trials, in combination with either a blockade of programmed cell death protein 1 (PD1)–programmed cell death 1 ligand 1 (PDL1) interaction or blockade of cytotoxic T lymphocyte antigen 4 (CTLA4). Owing to their roles in transcriptional regulation, DNA methyltransferase (DNMT) inhibitors and histone deacetylase (HDAC) inhibitors upregulate the expression of the antigen-presenting major histocompatibility complex (MHC) molecules, tumour antigens, and T-helper 1 (TH1)-type chemokines CXC-motif chemokine ligand 9 (CXCL9) and CXCL10. EZH2 inhibitors also upregulate the expression of TH1-type chemokines, which promote the tumour infiltration of cytotoxic CD8^+^ T-cells and natural killer (NK) cells, resulting in immune cell-dependent tumour regression. In addition, DNMT inhibitors increase double-stranded RNA (dsRNA) levels in cancer cells, creating a ‘viral mimicry’ and inducing a type I interferon (IFN) response. DNMT and HDAC inhibitors upregulate PDL1 levels in cancer cells, whereas bromodomain and extra-terminal (BET) inhibitors downregulate PDL1 levels. Moreover, HDAC inhibitors upregulate the expression of MICA (MHC class I polypeptide-related sequence A) and MICB, as well as the ligands of the activating receptor on NK cells (NKG2D), and promote tumour clearance by NK cells. TCR: T-cell receptor, PD1: programmed cell death protein 1, PDL1: programmed cell death 1 ligand 1, CTLA4: cytotoxic T lymphocyte antigen 4, DNMT: DNA methyltransferase, HDAC: histone deacetylase, MHC: major histocompatibility complex, TH1: T-helper 1, CXCL9: chemokines CXC-motif chemokine ligand 9, EZH2: Enhancer of zeste homolog 2, dsRNA: double-stranded RNA, IFN: interferon, BET: bromodomain and extra-terminal, MICA: MHC class I polypeptide-related sequence A, and NK: natural killer.

The process of histone acetylation relaxes the structure of chromatin, rendering it more vulnerable to the activity of transcription factors. Increased expression of HDACs, which is often accompanied by increased histone acetylation, is a hallmark of many types of cancer [201]. Tumour suppressor genes including p21 (CDKN1A) are transcribed into mRNA once HDACi are expressed (CDKN1A) [202]. HDACi have been demonstrated to be efficient in triggering the apoptotic mechanism of T-cells in several cancer types, both in vitro and in vivo [203,204,205]. HDACi have been shown to regulate MHC activity and costimulatory molecules (CD40) in a favourable manner [206,207]. In addition to its role in suppressing regulatory T-cells and promoting the attachment of the NK cell receptor (NKG2D) to ligands MICA and MICB, HDAC also regulates a pathway that includes the synthesis of tumour antigens and the antigen recruitment complex [208,209]. HDACi entinostat in combination with the aromatase inhibitor exemestane increases the suppression rate of myeloid-derived suppressor cells (MDSCs) and the activity of human leukocyte antigens-DR on monocytes while maintaining the CD8/CD4 T-cell ratio in a randomised Phase II study of hormone receptor-positive breast cancer. Figure 4 illustrates the important role of epigenetic inhibitors in MM immuno-oncology.

## 9. Conclusions and Future Directions

Epigenetics encompasses the interaction of DNA and histone proteins, but many uncertainties exist. Therefore, chromatin remains a critical therapeutic target. To enhance our comprehension of illness progression and to discover prospective therapy targets based on the molecular genesis of the disease, characterisation of the MM epigenetic landscape has emerged as the primary focus of ongoing research. Numerous studies on MM have revealed the roles of DNA methylation, histone modification, and non-coding RNAs in the development, progression, clonal heterogeneity, and therapy susceptibility of tumours. The goal of expanding our knowledge of the epigenetics of MM will eventually lead to the discovery of agents or their combinations that could be used in a precision therapeutic strategy to win the battle against MM. To eradicate MM totally, the discovery of an effective epigenetic inhibitor treatment will contribute to the basic understanding of the disease as well as the clinical capability to treat it.

## Figures and Tables

**Table 1:** DNA methylation targeting gene-specific in MM.

DNA Methylation/Gene	Effects	References
Promoter/hypermethylation CDKN2A and CDKN2B	Activates cell cycle Enhances cell proliferation and disease progression	[49,50,51]
LAPTm5 gene	Loss of E3 gene activity Crucial in MM progression	[52]
CHD1	Suppresses cell adhesion Increases cell motility Promotes metastasis	[53,54]
Promoter/hypermethylation WNT signalling pathway inhibitor genes (SFR1, SFR2, SFR4, SFR5, APC, WIF1, and DKK3)	Activates WNT signal	[55,56]
Promoter/hypermethylation DCC	Involved in cell migration Improves MM cells’ sensitivity to bortezomib	[56]
Promoter/hypomethylation Notch ligand JAG2	Increases expression growth factor IL-6 Enhances cell proliferation	[57]
Hypomethylation ATP binding cassette transporter ABCG2-hypomethylation	Involved in drug resistance	[58]
Promoter/hypomethylation CpG1 within the enhancer of MYBPHL	Promotes myelomagenesis	[59]
Promoter/hypermethylation DAPK1	Involved in early event MM pathogenesis Worst therapeutic response and short survival	[60,61,62]
Reduced DNA methylation Gene bodies at the loci of PRKCE, MGMT, FHIT, and WWOX	Poor survival Enhances MAF expression	[63]
Low methylation CXCR4 and NFKB1	Prolongs PFS and OS in relapsed patients following bortezomib treatment	[64]
Hypermethylation GPX3, RBP1, SPARC, and TGFB1	Aggressive phenotype of MM cells Extremely short OS	[65]
Homeobox genes	Osteogenic differentiation MM bone disease	[42]

CDKN2A: cyclin dependent kinase inhibitor 2A, CDKN2B: cyclin dependent kinase inhibitor 2B, LAPTm5: transmembrane-5, CHD1: chromodomain-helicase DNA-binding 1, WNT: wingless-related integration site, SFR: SWI5 dependent homologous recombination repair protein, APC: APC regulator of WNT signalling pathway, WIF1: Wnt inhibitory factor 1, DKK3: dickkopf WNT signalling pathway inhibitor 3, DCC: DCC netrin 1 receptor, JAG2: jagged canonical notch ligand 2, ABCG2: ATP-binding cassette transporter G2, MYBPHL: myosin binding protein H-like, DAPK1: death associated protein kinase 1, CDKN2A: cyclin-dependent kinase inhibitor 2A, PRKCE: protein kinase C epsilon type, MGMT: methylguanine methyltransferase, FHIT: fragile histidine triad diadenosine triphosphatase, WWOX: WW domain containing oxidoreductase, CXCR4: C-X-C motif chemokine receptor 4, NFKB1: nuclear factor kappa B subunit 1, GPX3: glutathione peroxidase 3, RBP1: retinol binding protein 1, SPARC: secreted protein acidic and cysteine rich, TGFB1: transforming growth factor beta-1, MAF: MAF BZIP transcription factor, IL-6: interleukin 6, PFS: progression-free survival, and OS: overall survival.

**Table 2:** The types of epigenetic inhibitors administered to patients with MM.

Epigenetic Inhibitors	Mechanisms	References
DNMT inhibitors		
Azacytidine	Destructs proteosome DNMT and decondenses chromatin Enhances necrosis via oxidative stress	[137] [138]
5-aza-2′deoxycytidine/decitabine	Damages DNA via gamma-H2AX foci formation G0/G1- or G2/M-phase arrest and caspase-mediated apoptosis Activates DDR	[139]
HDAC inhibitor		
Entinostat	HDACi Class I, effectively inhibits HDAC1 and HDAC3 Induces apoptosis via the downregulation of erbB3 expression Loosens the chromatin conformation and exposes DNA structure to destructive agents	[140] [141] [114]
Panobinostat (LBH-589)	HDACi Class I, II, and IV Dysregulates canonical Wnt signalling and key player β-catenin Reactivates cancer-suppressed genes and promotes cell death Significant toxicity across all HDAC classes	[142] [143] [144]
Vorinostat	HDACi class I and II Enhances cancer cell-cycle arrest and apoptosis Upregulates the E-cadherin gene and is less toxic than monotherapy or combination therapy	[115] [145]
Romidepsin	Cyclic tetrapeptide HDAC inhibitor HDACi Class 1, Class 2, and Class 6 Enhances cancer suppressor genes p21 and p53 Suppresses antiapoptotic molecules, e.g., Bcl-2, Bcl-XL, BAX, and MCL-1 Initiates apoptosis in a dose-dependent fashion Enhances p53, agitates the function of HSP90, tubulin, and the endoplasmic reticulum, and forms aggresomes Induces cell-cycle arrest (via the p21 and AKT pathways)	[146] [147] [148,149] [150]
ACY-241 (citarinostat)	Second-generation selective HDAC6 inhibitor More selective (13 to 18-fold) HDAC6 in comparison to HDAC1-3 Promotes higher serum concentrations Higher rating of HDAC inhibition including HDAC1/2 Alternative for potent and well-tolerated oral HDAC inhibitor	[151] [152]
ACY-1215 (ricolinostat)	Enhances α-tubulin acetylation and accumulation of ubiquitinated proteins Reduces the inhibition of class I HDACs Lower toxicity than nonselective HDAC inhibitors	[152,153]
Trichostatin A (TSA)	HDACi class I and II Effectively inhibits cell proliferation and initiates cell cycle arrest and apoptosis Sensitises TNF-related apoptosis-inducing factor (TRAIL)-resistant cells by suppressing the antiapoptotic BCL2 proteins Decreases expression of MM proliferation-associated factors	[154] [155] [156]

DNMT: DNA methyltransferase, H2AX: H2A.X variant histone, DDR: DNA damage response and repair, HDAC: histone deacetylase, HDACi: histone deacetylase inhibitors, erbB3: erb-b2 receptor tyrosine kinase 3, Bcl-2: B-cell lymphoma-2, Bcl-XL: B-cell lymphoma-extra-large, BAX: BCL2 associated X, MCL-1: myeloid leukaemia 1, HSP90: heat shock protein 90, p21: inhibitor of a cyclin-dependent kinase, AKT: protein kinase B, TRAIL: TNF-related apoptosis-inducing factor, and BCL2: B-cell lymphoma-2.

## Data Availability

Data are contained within the article.
